# Analysis of metabolite and strain effects on cardiac cross-bridge dynamics using model linearisation techniques

**DOI:** 10.3389/fphys.2023.1323605

**Published:** 2024-01-16

**Authors:** Julia H. Musgrave, June-Chiew Han, Marie-Louise Ward, Andrew J. Taberner, Kenneth Tran

**Affiliations:** ^1^ Auckland Bioengineering Institute, University of Auckland, Auckland, New Zealand; ^2^ Department of Physiology, University of Auckland, Auckland, New Zealand; ^3^ Department of Engineering Science and Biomedical Engineering, University of Auckland, Auckland, New Zealand

**Keywords:** cross-bridge kinetics, metabolites, cross-bridge strain, cardiac energetics, complex modulus, sinusoidal perturbation analysis

## Abstract

Multi-scale models of cardiac energetics are becoming crucial in better understanding the prevalent chronic diseases operating at the intersection of metabolic and cardiovascular dysfunction. Computationally efficient models of cardiac cross-bridge kinetics that are sensitive to changes in metabolite concentrations are necessary to simulate the effects of disease-induced changes in cellular metabolic state on cardiac mechanics across disparate spatial scales. While these models do currently exist, deeper analysis of how the modelling of metabolite effects and the assignment of strain dependence within the cross-bridge cycle affect the properties of the model is required. In this study, model linearisation techniques were used to simulate and interrogate the complex modulus of an ODE-based model of cross-bridge kinetics. Active complex moduli were measured from permeabilised rat cardiac trabeculae under five different metabolite conditions with varying ATP and P_i_ concentrations. Sensitivity to metabolites was incorporated into an existing three-state cross-bridge model using either a direct dependence or a rapid equilibrium approach. Combining the two metabolite binding methods with all possible locations of strain dependence within the cross-bridge cycle produced 64 permutations of the cross-bridge model. Using linear model analysis, these models were systematically explored to determine the effects of metabolite binding and their interaction with strain dependence on the frequency response of cardiac muscle. The results showed that the experimentally observed effects of ATP and P_i_ concentrations on the cardiac complex modulus could be attributed to their regulation of cross-bridge detachment rates. Analysis of the cross-bridge models revealed a mechanistic basis for the biochemical schemes which place P_i_ release following cross-bridge formation and ATP binding prior to cross-bridge detachment. In addition, placing strain dependence on the reverse rate of the cross-bridge power stroke produced the model which most closely matched the experimental data. From these analyses, a well-justified metabolite-sensitive model of rat cardiac cross-bridge kinetics is presented which is suitable for parameterisation with other data sets and integration with multi-scale cardiac models.

## 1 Introduction

The interactions between cellular energy metabolites and tension development in the heart are crucial for understanding the effects of prevalent metabolic diseases on cardiac health. The key contractile properties of the heart originate at the sub-cellular level, where acto-myosin cross-bridges transduce chemical energy from the hydrolysis of adenosine triphosphate (ATP) into mechanical force. To simulate and interrogate the effects of disease-induced changes in cellular metabolic state on cardiac force production across cell, tissue and organ scales, a computationally efficient model of cardiac cross-bridge kinetics that is sensitive to changes in metabolite concentrations is required. Models of cross-bridge kinetics which are simplified for multi-scale applications do currently exist (Razumova et al., 1999; Rice et al., 2008; Land et al., 2017; Filipovic et al., 2022), but there is a lack of analysis to determine how the assignment of metabolite dependence within the cross-bridge cycle affects the mechanical properties of these simplified models.

Cross-bridge kinetics are often characterised using complex modulus data where the force response of permeabilised muscles is normalised to small-amplitude sinusoidal length perturbations at a range of frequencies (Tanner et al., 2011). It is an information-rich measurement that captures, and has long been used to characterise, cross-bridge kinetics (Machin and Pringle, 1960; Kawai and Brandt, 1980; Palmer et al., 2007). Additionally, the active complex modulus reflects the influence of ATP and its hydrolysis products on the cross-bridge cycle and has thus been instrumental in developing detailed models outlining the biochemical steps in this cycle (Kawai et al., 1993). Our recent work has demonstrated that we can use model linearisation techniques to recapitulate the complex modulus of cardiac muscle by adding together appropriately weighted transfer functions associated with sarcomere length, sarcomere velocity and cross-bridge strain dependencies (Musgrave et al., 2022). Applying these linearisation techniques to cross-bridge models that are sensitive to ATP and inorganic phosphate (P_i_) will now allow identification of the key properties of the model that can account for the specific effects of these metabolites.

For a cross-bridge model to be able to respond to changes in metabolic state, it needs to be sensitive to the concentrations of cellular metabolites. While the incremental biochemical steps involving metabolites and cross-bridge filaments have been well studied (Houdusse and Sweeney, 2016; Walklate et al., 2016), a lack of consensus remains regarding the most appropriate way to mathematically distil this knowledge when integrating these effects into a reduced-state cross-bridge model. One method, used by Tran et al. (2010), is to include ATP and P_i_ concentrations into pseudo-first-order rate constants. In other words, these metabolites are combined into an existing step in the three-state scheme. Providing some additional complexity, Tewari et al. (2016) employed a rapid equilibrium approach to incorporate ATP and P_i_ concentrations. This method includes a sub-state within the model that is assumed to be rapid in comparison to the main steps, allowing for it to be simplified in the mathematical formulation.

Within the cross-bridge cycle, regulation of transition rates by metabolite binding may involve interactions with strain dependent mechanisms. Strain dependence is an essential component in cross-bridge theory and describes changes in the likelihood of binding or unbinding based on the amount of strain in a cross-bridge (Huxley, 1957). As both strain and metabolite dependencies are implemented through model transition rates, the strain dependence may be altered by changes in metabolite concentrations. Strain dependence is typically implemented for transition rates that describe an unbinding or detachment step, although published models have incorporated strain dependence in a range of transition rates (Rice et al., 2008; Tewari et al., 2016; Land et al., 2017). Our previous work also showed that the complex modulus could be recapitulated by placing strain dependence at a number of different transition rates within the cross-bridge cycle (Musgrave et al., 2022). However, incorporating the effect of metabolite dependence should give us more information about these transition rates and allow us to hone in on which of these is most appropriate.

In this study, we aim to develop a parsimonious model of cross-bridge mechanics, based on ordinary differential equations (ODEs), which incorporates the effect of changing ATP and P_i_ concentrations. While many iterations are typically considered in the development of any model, linearisation techniques offer the unique advantage of facilitating a systematic and quantitative analysis of a large number of model permutations due to their computational efficiency. We expect to find multiple combinations of strain and metabolite dependence assignments which can reproduce the general response of the cardiac complex modulus to changes in ATP and P_i_ concentrations. However, a systematic comparison of these different model permutations through the linearisation approach will allow us to quantify the best model fit to the data as well as explore the mechanisms that underpin the differences between these models. This method enables us to ultimately develop the simplest model of cardiac cross-bridge kinetics which incorporates the effect of changes in metabolite concentrations to facilitate multi-scale modelling of energetic state in the heart. Additionally, linearisation offers a simple way to examine the interacting dependencies in the model (Campbell et al., 2001), providing justifications for the underlying mechanisms.

## 2 Materials and methods

### 2.1 Experimental methods

Measurements of passive and total complex moduli were collected from a cohort of permeabilised rat ventricular trabeculae in order to produce a data set of active complex moduli across a range of ATP and P_i_ concentrations. Total complex moduli are most commonly measured or reported (Fukagawa et al., 2005; Palmer et al., 2013), despite the active complex modulus providing much clearer information about cross-bridge activity (Musgrave et al., 2022). This measurement eliminates the effect of the passive stiffness measured in low Ca^2+^ (relaxing) solutions and isolates the active properties which are only present following activation of the muscle with high Ca^2+^ solutions.

#### 2.1.1 Solutions

All of the following experimental solutions were adjusted with KCl to produce a final ionic strength of 180 mM and with KOH to produce a pH of 7.0 at the stated temperature. The final concentrations of each metal, ligand, and metal-ligand complex were calculated using version 2.5 of the MaxChelator computer program (Bers et al., 2010). Permeabilisation solution was used at 4°C and contained: 7 mM EGTA, 20 mM imidazole, 4 mM MgATP, 1 mM Mg^2+^, 10 μg/mL leupeptin, 30 mM BDM, 1 % v/v Triton X-100, 50 % v/v glycerol. Relaxing solution was used at room temperature, had a pCa of 9.0 and contained: 7 mM EGTA, 20 mM imidazole, 5 mM MgATP, 1 mM Mg^2+^, 1 mM P_i_, 14.5 mM CrP. Pre-activating solution was the same as Relaxing solution, except with EGTA reduced to 0.5 mM and 6.5 mM HDTA added. Baseline activating solution was Relaxing solution with Ca^2+^ concentration increased to pCa 4.5. The varied P_i_ solutions were Baseline activating solution with 0 mM or 5 mM P_i_. The varied ATP solutions were Baseline activating solution with 0.1 mM or 1 mM MgATP. The five activating solutions are summarised in Table 1.

**TABLE 1:** ATP and P_i_ concentrations of the five activating solutions used for this study.

Condition	[MgATP] (mM)	[P_i_] (mM)
Baseline	5	1
Low ATP	1	1
Super-low ATP	0.1	1
No P_i_	5	0
High P_i_	5	5

#### 2.1.2 Sample preparation

Trabeculae (*n* = 11) were dissected from the right ventricle of male Wistar rats (ethics approval: AEC22653), following previously described procedures (Dowrick et al., 2021). Following dissection, trabeculae were placed in 4°C permeabilisation solution for approximately 20 h. Permeabilised trabeculae were then rinsed with relaxing solution before beginning the experiment.

#### 2.1.3 Experimental protocol

Experiments were conducted on the Muscle Mechanometer, a previously-described custom device (Choi et al., 2021; Musgrave et al., 2023). Permeabilised trabeculae were mounted in a tissue bath filled with relaxing solution between a voice-coil motor and stainless-steel cantilever. A 40x Nikon inverted microscope was used to image sarcomeres as the muscle was gradually stretched to a sarcomere length of 2.2 *μ*m. Muscle diameters in the short and long axes were measured to convert the measured force to stress (force per cross-sectional area), assuming an elliptical cross-section. All experiments were performed at room temperature.

For each muscle, a passive complex modulus measurement was made while the muscle was in relaxing solution (pCa 9.0). Next, the muscle was switched rapidly ( < 1 s) into a bath containing preactivating solution for 2 min. Following this, the muscle was switched again into a bath with one of the activating solutions from Table 1. The trabecula was held at a constant length until the generated stress reached a steady state. Here, a total (active + passive) complex modulus measurement was made before returning the muscle to the relaxing solution. This process was repeated for each of the five activating solutions, which were not presented in any fixed order.

Complex modulus measurements were conducted by first recording muscle stress under sinusoidal length perturbations of 0.25 % of muscle length across 17 frequencies logarithmically spaced between 0.1 Hz and 100 Hz (exclusive). The complex modulus was then calculated at each measurement frequency as the fast Fourier transform of stress divided by the fast Fourier transform of normalised muscle length. Finally, the active complex modulus under each set of metabolite conditions was found by subtracting the passive complex modulus for the given muscle from the total complex modulus measured under full activation.

Means and standard errors (SEM) were calculated for the steady-state stress and at each of the 17 frequencies for the complex modulus, under each of the five metabolite conditions measured.

### 2.2 Mathematical model

#### 2.2.1 Baseline model

The cross-bridge model used to test different assignments of metabolite and strain dependence is based on that which was presented in our previous work (Musgrave et al., 2022). This is a three-state stiffness-distortion model (Razumova et al., 1999) which assumes that cross-bridges are fully activated with Ca^2+^. The active stress (force normalised to cross-sectional area) produced by this model is:

$$F=K\left(Bx_{B}+Cx_{C}\right),$$ (1)

where *F* is the active stress generated by attached cross-bridges; *B* and *C* are state variables representing the proportion of cross-bridges in each of the two attached states, pre- and post-power stroke, respectively; *x*_B_ and *x*_C_ are state variables representing the mean values of cross-bridge strain in the pre-power stroke state **B** and post-power stroke state **C**, respectively; *K* is a spring constant representing the stiffness of the collective myosin heads. A set of coupled ordinary differential equations (ODEs) describes each of the four state variables in Eq. 1. The rates of change of the proportion of cross-bridges in states **B** and **C** are derived from the law of mass action:

$$\frac{dB}{dt}=k_{1}A-\left(k_{-1}+k_{2}\right)B+k_{-2}C,$$ (2)

$$\frac{dC}{dt}=k_{2}B-\left(k_{-2}+k_{3}\right)C+k_{-3}A,$$ (3)

where *k*_1_, *k*_2_, *k*_3_, *k*_−1_, *k*_−2_ and *k*_−3_ are the transition rates between cross-bridge states. The proportion of cross-bridges in state **A** is given by the principle of conservation:

$$A=Z\left(L\right)-B-C.$$ (4)

The proportion of all possible cross-bridges which are able to bind for a given sarcomere length is given by *Z* in Eq. 4. The equation for *Z* therefore describes the length-dependence in the model:

$$Z\left(L\right)=\left\{\begin{array}{cc} 1+\phi_{l}\left(\frac{L}{L_{max}}-1\right)\; & L<L_{max},\\ 1\; & L_{max}\leq L\leq 2.4, \end{array}\right.$$ (5)

where *L* is the sarcomere length, *ϕ*_l_ is a unitless parameter which governs the slope of the force-length relationship. *L*_max_ is the sarcomere length at which the maximum number of binding sites are available for cross-bridge formation and was taken as 2.3 *μ*m (Rice et al., 2008). The model is not defined for lengths greater than 2.4 *μ*m, as stretching beyond this length (into the descending arm of the force-length relationship) can lead to irreversible muscle damage.

The rates of change for the mean cross-bridge strains in the attached states are governed by the following ODEs:

$$\frac{dx_{B}}{dt}=-\frac{\phi_{x}}{B}\left(k_{1}A+k_{-2}C\right)x_{B}+\phi_{v}\frac{dL}{dt},$$ (6)

$$\frac{dx_{C}}{dt}=-\frac{\phi_{x}}{C}\left(k_{2}B+k_{-3}A\right)\left(x_{C}-x_{C0}\right)+\phi_{v}\frac{dL}{dt},$$ (7)

where *ϕ*_x_ is a unitless parameter governing the strain dependence, *ϕ*_v_ is a unitless parameter governing the sarcomere velocity dependence and *x*_C0_ = 0.01 *μ*m, the strain associated with the power stroke, and is the steady-state value of *x*_C_.

#### 2.2.2 Linearisation

**FIGURE 1:**
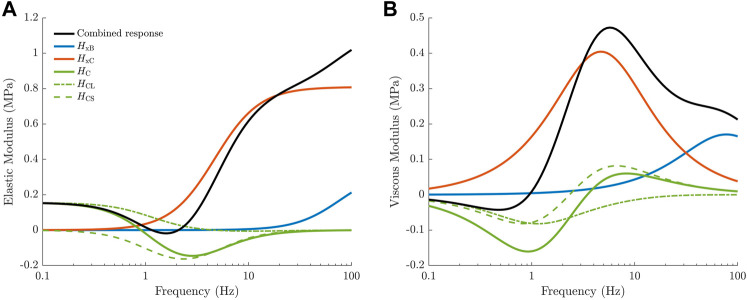
Transfer functions comprising the linearised cross-bridge model, taken from Musgrave et al. (2022). The elastic **(A)** and viscous **(B)** moduli of the overall complex modulus (black) are separated into three distinct components based on the transfer functions of the linearised model. Each transfer function reflects the behaviour of physiological cross-bridge properties under the length perturbation protocol. Cross-bridge strain produces high-pass frequency responses due to the influence of sarcomere velocity ( $H_{x_{B}}$ , blue; $H_{x_{C}}$ , red). The proportion of post-power stroke cross-bridges (*H*_C_, green), is influenced by two model properties. The influence of sarcomere length on the available cross-bridges (dash-dot line) produces a static modulus, while the influence of cross-bridge strain on the occupancy of attached states (dashed line) produces a negative band-pass filter response in the elastic modulus.

Data fitting and analysis of the cross-bridge model was performed using a linearised version of the full ODE model. The linearised model fully captures the complex modulus behaviour of the full model (Figure 1). This approach was developed by Razumova and Campbell (Razumova et al., 1999; Campbell et al., 2001) and its application to this model is described in more detail in Musgrave et al. (2022). Briefly, because the complex modulus measures small perturbations around a reference state, it can be approximated by performing a linearisation of the active force function (Eq. 1), followed by a Fourier transform into the frequency domain:

$$\frac{dF}{dL}\left(j\omega \right)=K\left(B_{0}H_{x_{B}}\left(j\omega \right)+C_{0}H_{x_{C}}\left(j\omega \right)+x_{C0}H_{C}\left(j\omega \right)\right),$$ (8)

where $\frac{dF}{dL}\left(j\omega \right)$ is the complex modulus of the model (around sarcomere length 2.2 *μ*m). A subscript 0 refers to the steady-state reference value of a given model variable. $H_{x_{B}}\left(j\omega \right),H_{x_{C}}\left(j\omega \right)$ and *H*_C_(*jω*) are the transfer functions of the state variables *x*_B_, *x*_C_ and *C*, which represent their behaviour under incremental length perturbations across the frequency spectrum. The expressions for these algebraic transfer functions are found by taking a first-order Taylor series expansion of each of the state variable ODEs (Eqs 2, 3, 6 and 7). In general form the Taylor expansions are:

$${\dot{y}}_{i}=\underset{j}{\sum }\partial_{i}^{j}y_{j}+\partial_{i}^{u}u,$$ (9)

where ${\dot{y}}_{i}$ represents the incremental deviation of state variable *i* around its reference value, *u* represents an incremental deviation of the input parameter, sarcomere length, around its reference value and $\partial_{i}^{j}$ is the partial derivative of the *i*th ODE with respect to the *j*th state variable (or with respect to the input parameter for $\partial_{i}^{u}$ ), where all derivatives are evaluated at the reference state. Indices *i* and *j* both take four values, each representing the four state variables, *B*, *C*, *x*_B_ and *x*_C_.

The four equations in the form of Eq. 9 (each representing the ODE describing *B*, *C*, *x*_B_ or *x*_C_) are Fourier transformed, divided by the incremental length perturbation *u* and rearranged to form the three transfer functions in Eq. 8. Thus, these transfer functions are influenced by the partial derivatives of the ODEs, reflecting the interdependencies between state variables and between state variables and the input parameter. For the baseline model described in Section 2.2.1, the transfer functions have the following forms:

$$H_{x_{B}}=\frac{\partial_{x_{B}}^{u}j\omega }{j\omega -\partial_{x_{B}}^{x_{B}}};$$ (10)

$$H_{x_{C}}=\frac{\partial_{x_{C}}^{u}j\omega }{j\omega -\partial_{x_{C}}^{x_{C}}};$$ (11)

$$H_{C}=H_{CL}+H_{CS}$$ (12)

where *H*_CL_ arises from length-dependent effects in the model and *H*_CS_ arises from strain dependence.

$$H_{CL}=\frac{\partial_{B}^{u}\partial_{C}^{B}}{\left(j\omega -\partial_{C}^{C}\right)\left(j\omega -\partial_{B}^{B}\right)-\partial_{C}^{B}\partial_{B}^{C}}$$ (13)

*H*_CS_ will be described in Section 2.2.4 when strain dependence is introduced to the model.

#### 2.2.3 Incorporation of ATP and P_i_ dependence

**FIGURE 2:**
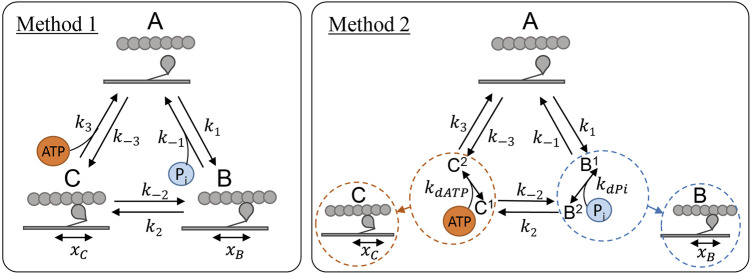
Cross-bridge model schematics showing the two different methods of incorporating ATP and P_i_ dependence into the model described in Section 2.2.1. Left (Method 1): Direct dependence of the metabolites where P_i_ concentration directly scales the detachment rate, *k*_−1_, and ATP concentration scales the detachment rate *k*_3_, as in Tran et al. (2010). Right (Method 2): Indirect dependence where a *k*_*d*_ parameter is included for each metabolite to represent the rapid binding which takes place during the B and C states for P_i_ and ATP respectively.

We explored two methods for incorporating the effect of ATP and P_i_ concentration into the existing three-state cross-bridge model. In Method 1, direct dependence (Figure 2, left panel) was implemented as in Tran et al. (2010), by considering P_i_ as a reactant in the unbinding reaction (counterclockwise) between state **B** and state **A** and ATP as a reactant in the unbinding reaction (clockwise) between state **C** and state **A**. In this scheme the magnitude of these reaction rates are scaled directly by the concentration of the relevant chemical species such the effective rates for each reaction are:

$$k_{-1}=k_{-1}^{\prime }\left[P_{i}\right],$$ (14)

$$k_{3}=k_{3}^{\prime }\left[ATP\right],$$ (15)

where $k_{-1}^{\prime }$ and $k_{3}^{\prime }$ are the initial rates (s^−1^⋅mM^−1^) for *k*_−1_ and *k*_3_, before metabolite scaling and [P_i_] and [ATP] are the concentrations of P_i_ and ATP.

In Method 2, indirect dependence (Figure 2, right panel) was implemented under the assumption of rapid equilibrium. Under this scheme, we assume that the binding of ATP or P_i_ takes place in between the transitions between the three major mechanical states and is comparatively rapid. This scheme involves an extra parameter for each of the metabolite bindings, a *k*_d_ value, and both transition rates leaving states **B** and **C** are scaled by a non-linear function of the concentration of the metabolite:

$$k_{-1}=k_{-1}^{\prime }\frac{\left[P_{i}\right]}{k_{dPi}+\left[P_{i}\right]}$$ (16)

$$k_{2}=k_{2}^{\prime }\frac{k_{dPi}}{k_{dPi}+\left[P_{i}\right]}$$ (17)

$$k_{-2}=k_{-2}^{\prime }\frac{k_{dATP}}{k_{dATP}+\left[ATP\right]}$$ (18)

$$k_{3}=k_{3}^{\prime }\frac{\left[ATP\right]}{k_{dATP}+\left[ATP\right]}$$ (19)

where $k_{i}^{\prime }$ indicates the initial rate (s^−1^) for *k*_*i*_, before metabolite scaling, *k*_d_ is the dissociation constant for either P_i_ or ATP and [P_i_] and [ATP] are the concentrations of P_i_ and ATP.

While placement of ATP and P_i_ binding steps at other locations were considered, the two methods we examined in Figure 2 were the most plausible for a three-state model, as supported by previous modelling studies (Tran et al., 2010; Offer and Ranatunga, 2020).

#### 2.2.4 Incorporation of strain dependence

In cross-bridge models, strain dependence is typically incorporated using exponential functions. For example, in our previously-published model (Musgrave et al., 2022) the detachment rate, *k*_3_, increases exponentially with increasing strain in state **C**:

$$k_{3}=k_{3}^{\prime }e^{\phi_{s}\left(x_{C}-x_{C0}\right)},$$ (20)

where *ϕ*_s_ is a scaling parameter governing the strain dependence and $k_{3}^{\prime }$ indicates the steady-state value for *k*_3_, without any strain.

The inclusion of strain dependence augmented the ‘dip’ in the elastic modulus and produced a negative viscous modulus; both are characteristic features of the cardiac muscle frequency response. These features are theoretically reproducible with any manoeuvres which can reduce the number of cross-bridges in state **C** with increasing strain. We chose to systematically explore the effect of incorporating exponential strain dependence on each of the transition rates in the cross-bridge model, as the effect of metabolite concentration on strain dependence may help to narrow down these options. The strain-dependent component of *H*_C_ is a negative band-pass function, as described by.

$$H_{CS}=\frac{\partial_{C}^{B}\left(\partial_{B}^{x_{B}}H_{x_{B}}+\partial_{B}^{x_{C}}H_{x_{C}}\right)+\left(\partial_{C}^{x_{B}}H_{x_{B}}+\partial_{C}^{x_{C}}H_{x_{C}}\right)\left(j\omega -\partial_{B}^{B}\right)}{\left(j\omega -\partial_{C}^{C}\right)\left(j\omega -\partial_{B}^{B}\right)-\partial_{C}^{B}\partial_{B}^{C}}$$ (21)

Note that the partial derivatives $\partial_{B}^{x_{B}}$ , $\partial_{C}^{x_{B}}$ , $\partial_{B}^{x_{C}}$ and $\partial_{C}^{x_{C}}$ represent the effect of strain on attached cross-bridge proportion and may have a value of zero, depending on which strain dependence is implemented.

The five different placements of strain dependence we considered are presented in Table 2. The equations were each formulated to augment the elastic modulus dip, by providing a negative band-pass shape to *H*_CS_. Mathematically, this is achieved by constraining the signs of the relevant partial derivatives among $\partial_{B}^{x_{B}}$ , $\partial_{C}^{x_{B}}$ , $\partial_{B}^{x_{C}}$ and $\partial_{C}^{x_{C}}$ to ensure the numerator of Eq. 21 is negative. In a mechanistic sense, this corresponds to strain dependencies which encourage a reduction in the number of cross-bridges in state **C** with strain. Strain dependence on *k*_−3_ was not considered due to the negligible size of this baseline rate.

**TABLE 2:** Description of strain dependence added to each of the rates in the cross-bridge model. The effect of strain on each rate was selected to cause a reduction of *C* with strain. *ϕ*_s*i*_ is a parameter which governs the extent of strain dependence on the rate *k*_*i*_.

Rate	Effect of strain on the rate	Rate equation
*k*_1_	Decreases with *x*_B_	$k_{1}=k_{1}^{\prime }e^{-\phi_{s1}x_{B}}$
*k*_−1_	Increases with *x*_B_	$k_{-1}=k_{-1}^{\prime }e^{\phi_{s-1}x_{B}}$
*k*_2_	Decreases with *x*_B_	$k_{2}=k_{2}^{\prime }e^{-\phi_{s2}x_{B}}$
*k*_−2_	Increases with *x*_C_	$k_{-2}=k_{-2}^{\prime }e^{\phi_{s-2}\left(x_{C}-x_{C0}\right)}$
*k*_3_	Increases with *x*_C_	$k_{3}=k_{3}^{\prime }e^{\phi_{s3}\left(x_{C}-x_{C0}\right)}$

#### 2.2.5 Cross-bridge insensitive stiffness

In order to fit to the complex modulus across the range of different metabolite concentrations, we found it was necessary to add an additional stiffness component to the model (see Discussion). This stiffness is present in the active model, but does not scale with the proportion of attached cross-bridges, hence it was referred to as cross-bridge insensitive stiffness. We used a simple linear spring model to represent the corresponding stress, *F*_s_:

$$F_{s}=\left\{\begin{array}{cc} K_{s}\left(L-1.9\right)\; & L>1.9,\\ 0\; & L\leq 1.9, \end{array}\right.$$ (22)

where *K*_s_ is the cross-bridge insensitive stiffness and *F*_s_ is only generated beyond the resting length of 1.9 *μ*m. The addition of *F*_s_ to the model (Eq. 1) results in a constant stiffness component (*K*_s_) in the complex modulus model, such that Eq. 8 becomes:

$$\frac{dF}{dL}\left(j\omega \right)=K\left(B_{0}H_{x_{B}}+C_{0}H_{x_{C}}+x_{C0}H_{C}\right)+K_{s}$$ (23)

### 2.3 Model analysis

To systematically determine the effects and improvements in model fitting to the data associated with different combinations of metabolite binding complexity and strain placements, we tested 64 permutations of the model. These consisted of 16 different assignments of strain dependence combined with four different assignments of metabolite dependence. The 16 different assignments of strain dependence were: no strain dependence, strain dependence on each of the five rates described in Table 2 and strain dependence on every combination of pairs of these five rates. Combinations of three or more strain dependencies were not considered as two strain dependencies are typically the limit for three-state cross-bridge models (Razumova et al., 1999; Rice et al., 2008; Land et al., 2017). The four assignments of metabolite dependence came from the possible combinations of both methods of metabolite dependence presented in Figure 2. Each of ATP and P_i_ were incorporated using either Method 1 or Method 2.

Testing 64 permutations of the model was made possible by the significant computation time saved by linearising the model. The analytical solution for the complex modulus of the linearised model was computed several orders of magnitude faster than the full model, which requires a numerical solver.

#### 2.3.1 Model fitting protocol

For each of the 64 permutations, the linearised model of the complex modulus was solved analytically to find the best fit to all of the complex modulus data measured. The number of model parameters depended on the specific permutation. The minimum number of parameters (for the version with no strain dependence and direct metabolite dependence) was 10 and the maximum number of parameters (for versions with strain dependence on two rates and *k*_d_ values for both metabolites) was 14. The particle swarm algorithm was used in MATLAB (The MathWorks, Inc) to find the values of these parameters which minimised the following objective function.

$$OBJ\left(p\right)=\frac{1}{N_{c}}\overset{N_{c}}{\underset{i=1}{\sum }}\left(RMSE^{i}\left(p\right)+F_{0,err}^{i}\left(p\right)\right),$$ (24)

where *p* is the set of variable parameters to be optimised, *N*_c_ = 5, the number of experimental conditions being simulated (Table 1), *RMSE* ^*i*^(*p*) is the root-mean-square error in the complex modulus prediction under parameter set *p* and condition *i* and $F_{0,err}^{i}\left(p\right)$ is the error in the steady-state force production under parameter set *p* and condition *i*.

$$RMSE^{i}\left(p\right)=\sqrt{\frac{1}{2N_{f}}\overset{N_{f}}{\underset{j=1}{\sum }}\left({\left(E_{m}^{j}\left(p\right)-E_{d}^{j}\right)}^{2}+{\left(V_{m}^{j}\left(p\right)-V_{d}^{j}\right)}^{2}\right)},$$ (25)

where *N*_f_ = 17, the number of frequencies at which the complex modulus was collected, $E_{m}^{j}\left(p\right)$ and $V_{m}^{j}\left(p\right)$ are the elastic and viscous moduli predicted by the model under parameter set *p* at frequency *j*, $E_{d}^{j}$ and $V_{d}^{j}$ are the elastic and viscous moduli measurements at frequency *j*.

$$F_{0,err}^{i}\left(p\right)=\left\{\begin{array}{cc} |F_{0,m}\left(p\right)-F_{0,d}|-SE,\; & |F_{0,m}\left(p\right)-F_{0,d}|>SE\\ 0,\; & |F_{0,m}\left(p\right)-F_{0,d}|\leq SE \end{array}\right.$$ (26)

where *F*_0,m_(*p*) is the steady-state stress predicted by the model under parameter set *p*, *F*_0,d_ is the mean active steady-state stress from the data set and *SE* is the standard error in the specific value of *F*_0,d_. This function was introduced to ensure that reasonable steady-state forces were described by the model (Musgrave et al., 2022) and was formulated such that it does not influence the objective function provided the steady-state force falls within the standard error in the mean value measured.

#### 2.3.2 Model validation

To validate the final model in the time domain, a series of numerical force redevelopment simulations were performed using the full ODE cross-bridge model at the five different metabolite concentrations used in model fitting. The simulation of cross-bridge detachment was achieved by increasing detachment rates and decreasing attachment rates 500 fold for 2 ms, following Rice et al. (2008). Transition rates between states **B** and **C** were also increased by 10 fold for this period to encourage cross-bridge detachment. Following this detachment phase, the simulated stress within the muscle was recorded until it reached a final steady-state stress. These simulations allowed the model steady-state stresses to be compared with our experimental data. They also offered a qualitative validation with existing literature reporting on changes in rates of force redevelopment at different ATP and P_i_ concentrations.

Model code is available at https://github.com/JuliaMusgrave/XBModel_2024_Rat.

## 3 Results

### 3.1 Experimental results

**FIGURE 3:**
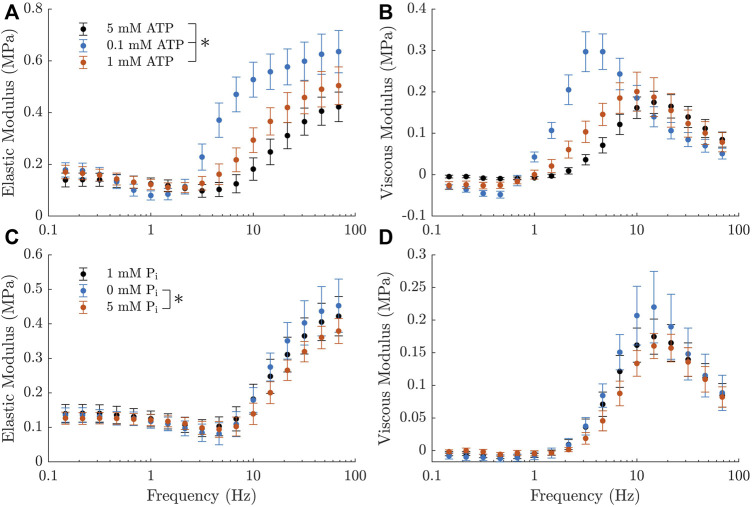
Active complex moduli measured in rat cardiac trabeculae across a range of metabolite concentrations, shown as mean ± SEM. **(A, B)** Elastic and viscous moduli as the ATP concentration is varied. P_i_ concentration is kept at 1 mM across these measurements. Curves shift upward and to the left with decreasing ATP concentration. **(C, D)** Elastic and viscous moduli as the P_i_ concentration is varied. ATP concentration is kept at 5 mM across these measurements. Curves shift upward with decreasing P_i_ concentration. There is also a subtle shift to the left with decreasing P_i_, which is much less evident than the effects of ATP, but can still be verified from the parameters of a simplified Kawai model (Kawai and Brandt, 1980) (*c* =11.7Hz for 0 mM P_i_, *c* =16.1Hz for 5 mM P_i_). The black markers represent the baseline measurement, which is common across both rows. * indicates statistical significance (*p* <0.05) on either component of the modulus using a two-way ANOVA model and comparing the effect of changing metabolite concentration with Tukey test. Significant differences were found between all three comparisons of ATP concentration and between 0 mM and 5 mM P_i_.

The elastic and viscous moduli measurements from our rat trabeculae experiments demonstrated the expected trends in response to changing metabolite concentrations. We observed that a decrease in ATP or P_i_ concentration resulted in an increased magnitude of the complex modulus and a leftward shift in frequency (Figure 3). Both of these effects are consistent with similar measurements from Kawai et al. (1993) and Palmer et al. (2013). Additionally, there was an increase in the active stress produced by the muscles at steady-state with reduced concentrations of each metabolite, as has also been previously reported (Godt and Nosek, 1989; Ebus et al., 2001; Hinken and McDonald, 2004). The active stresses measured under each metabolite condition were, in kPa: 21.8 ± 2.1 at baseline (5 mM ATP and 1 mM P_i_), 32.3 ± 2.4 at super-low ATP (0.1 mM), 27.7 ± 3.1 at low ATP (1 mM), 25.5 ± 2.9 at no P_i_, and 18.0 ± 1.6 at high P_i_ (5 mM).

All of these effects appeared to be more pronounced with changes in ATP concentration, suggesting that the muscles examined had a higher sensitivity to this metabolite. Additionally, there was a marked increase in the magnitude of the ‘dip’ in the elastic modulus with decreasing ATP concentration (Figure 3A, B). There was no visible effect on this feature with changing P_i_.

### 3.2 General effect of ATP and P_i_ on complex modulus models

**FIGURE 4:**
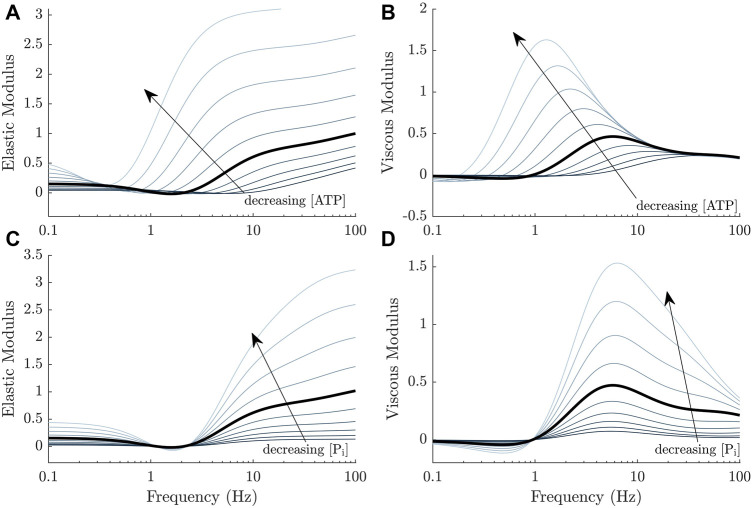
General effects of decreasing ATP and P_i_ concentrations on the complex moduli response of a cross-bridge model (Musgrave et al., 2022) where the metabolite dependencies are incorporated directly, using Method 1 (Figure 2, left panel). **(A, B)** The magnitude of both the elastic and viscous moduli increase and the curves shift to the left as the ATP concentration decreases. **(C, D)** The magnitude of both the elastic and viscous moduli also increase as the P_i_ concentration decreases. There is a more subtle shift in frequency compared to ATP, also to the left. This shift is more evident from the linearised model equations and at higher frequencies.

Before attempting to fit the set of 64 models to our complex modulus data, we first consider the effect of adding direct dependence of ATP and P_i_ to our previously published model (Musgrave et al., 2022), whose baseline frequency response is described in Figure 1. As hypothesised, we found that the simplest implementation of metabolite dependence produced the general effects of changing metabolite concentrations on the complex modulus. Figure 4 displays the effect of changing either ATP or P_i_ concentrations in our previous model (Musgrave et al., 2022), using direct dependence of these metabolites (Method 1). With no alterations to the model parameters (after accounting for the effect of baseline ATP and P_i_ values), most of the trends described in Section 3.1 were reproduced.

Lowering the ATP concentration decreases *k*_3_ (Eq. 15). This produced a higher steady-state active stress in the model, an increase in the magnitude of the complex modulus and shifted the frequency response to the left (Figure 4A, B). Lowering the P_i_ decreases *k*_−1_ and had similar effects. Examining how *k*_3_ and *k*_−1_ affect key components of the model and the transfer functions underlying the complex modulus, will reveal the underlying mechanisms of these trends. The following analyses are valid under the simplifying assumption that *k*_−3_ ≈ 0.

#### 3.2.1 Effect of detachment rates on attached cross-bridges

Decreasing detachment rates, whether it be via *k*_3_ or *k*_−1_, causes an increase in the steady-state proportion of cross-bridges in the post-power stroke state, **C**. We can show this mathematically by using the King-Altman rule (King and Altman, 1956) to derive the steady-state expression for *C*:

$$C_{0}=\frac{k_{1}k_{2}}{k_{3}\left(k_{1}+k_{2}+k_{-1}\right)+k_{1}k_{2}+k_{-2}k_{1}+k_{-2}k_{-1}}$$ (27)

*C*_0_ will increase with smaller values of *k*_3_, due to its presence solely in the denominator. Similarly, *C*_0_ will increase with smaller values of *k*_−1_ as it is also present in only the denominator Eq. 27. This mechanism, whereby slowed detachment kinetics results in more cross-bridges remaining in the attached state, is responsible for the following key effects associated with lowered metabolite concentrations.

1. Increased steady-state stress. As strain in state **B** is equal to zero at steady-state, Eq. 1 can be simplified to give the steady-state stress as:

$$F_{0}=KC_{0}x_{C0}+K_{s}\left(L_{0}-1.9\right)$$ (28)

2. Increased maximum elastic modulus. $H_{x_{C}}$ , the high-pass transfer function which corresponds to strain in state **C** is directly scaled by *C*_0_ (see Eq. 23). This means that *C*_0_ will affect the magnitude of this entire component, as well as the maximum elastic modulus.

$$E_{max}=K\phi_{v}\left(B_{0}+C_{0}\right)+K_{s}$$ (29)

Note that *B*_0_ also increases with decreasing values of *k*_−1_.

3. Increased static modulus. Simplification of the length dependent component of *H*_C_ reveals that the elastic modulus has the following magnitude as frequency approaches 0 Hz.

$$\begin{array}{cc} E_{0Hz} & =Kx_{C0}H_{CL}\left(0\right)+K_{s}\\ & =Kx_{C0}\frac{\partial_{B}^{u}\partial_{C}^{B}}{\partial_{C}^{C}\partial_{B}^{B}-\partial_{C}^{B}\partial_{B}^{C}}+K_{s}\\ & =Kx_{C0}\frac{\phi_{l}C_{0}}{L_{max}}+K_{s} \end{array}$$ (30)

#### 3.2.2 Effect of detachment rates on transfer functions

The leftward shifts in frequency in response to decreased metabolite concentrations are a direct effect of the role that *k*_3_ and *k*_−1_ play in the transfer functions. These rates come into play most directly with the two high-pass transfer functions, $H_{x_{B}}$ (Eq. 10) and $H_{x_{C}}$ (Eq. 11). The cutoff frequencies of these transfer functions, where the maximum slope in the elastic modulus occurs, correspond to the partial derivatives $\partial_{x_{B}}^{x_{B}}$ and $\partial_{x_{C}}^{x_{C}}$ :

$$\begin{array}{cc} \partial_{x_{B}}^{x_{B}} & =-\frac{\phi_{x}\left(A_{0}k_{1}+C_{0}k_{-2}\right)}{B_{0}}\\ & =-\phi_{x}\left(k_{2}+k_{-1}\right)\\ & =-\phi_{x}\left(k_{2}+k_{-1}^{\prime }\left[P_{i}\right]\right) \end{array}$$ (31)

$$\begin{array}{cc} \partial_{x_{C}}^{x_{C}} & =-\frac{\phi_{x}B_{0}k_{2}}{C_{0}}\\ & =-\phi_{x}\left(k_{3}+k_{-2}\right)\\ & =-\phi_{x}\left(k_{3}^{\prime }\left[ATP\right]+k_{-2}\right) \end{array}$$ (32)

Eq. 31 shows that the cutoff frequency of $H_{x_{B}}$ (blue curve in Figure 1) is influenced by the transition rates leaving state **B** and will decrease with decreasing P_i_ concentration, while Eq. 32 shows that the cutoff frequency of $H_{x_{C}}$ (red curve in Figure 1) is influenced by the transition rates leaving state **C** and will decrease with decreasing ATP concentration. These two effects are the key drivers of the leftward shift with decreasing metabolite concentrations. Additionally, the strain dependent transfer function, *H*_CS_, is proportional to either $H_{x_{B}}$ or $H_{x_{C}}$ (depending on the nature of the strain dependence) so this will contribute to the frequency shift, too.

### 3.3 Metabolite and strain effects: Comparison of different model permutations

**FIGURE 5:**
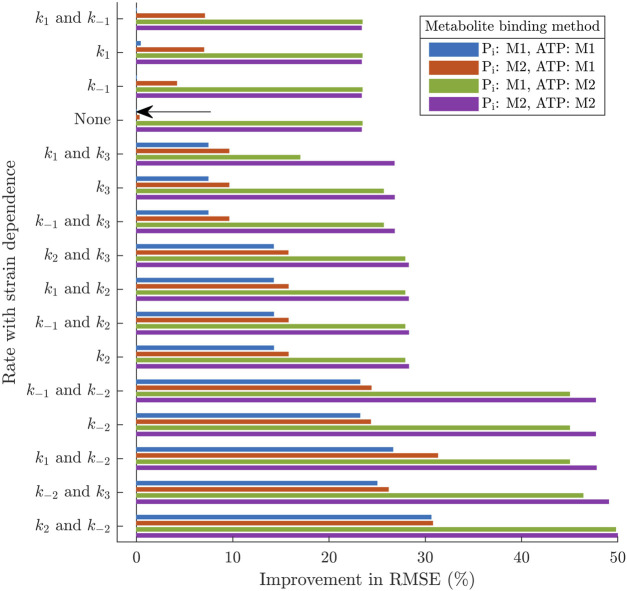
Percentage improvement in the Root-Mean-Sqaure-Error under the 64 different model permutations, in comparison to the simplest version of the model (1A, marked by an arrow). The simplest version of the model has no strain dependence, uses Method 1 for binding of both P_i_ and ATP and has 10 variable parameters. Each of the other versions of the model has 11–14 parameters.

While the previous section demonstrated the fundamental cross-bridge mechanisms that capture the general effects of changing ATP and P_i_ concentrations, in this next section, we assess the effects of metabolite binding complexity and its interaction with strain dependence. There was a total of 64 model permutations which resulted from 16 different combinations of strain dependencies and 4 different combinations of metabolite binding complexity. Since all permutations of the model found suitable steady-state stresses to minimise Eq. 26, the optimal value of the objective function simply reflected the average Root-Mean-Square-Error (RMSE) for the frequency response data across the five metabolite concentrations. Table 3 reveals that there were variations in the goodness of fit of the models to the data, depending on which permutation was used, and introduces a reference system for each of the permutations. The best RMSE value obtained was 1.72 % of the modulus range (Model 14D), while the worst were 3.49 % of the range (Models 1A, 3A, 7A). The key trends across the models are summarised in Figure 5 by examining the improvement in the RMSE value achieved for a given combination, compared to the simplest version of the model (1A). Model 1A has no strain dependence and has direct dependence (Method 1) on both ATP and P_i_.

**TABLE 3:** Normalised optimal objective function values found under a range of different assignments of strain and metabolite dependence (64 model permuations). M1 and M2 refer to Method 1 and Method 2 for metabolite binding (Figure 2). Each strain location is given a number and each metabolite assignment is given a letter to allow for the 64 permutations to be referenced more easily. The table values reflect the normalised Root-Mean-Square Error (%) in the complex modulus measurements, averaged over each of the metabolite conditions. The RMSE was normalised to the mean range across the five metabolite conditions, which was 556 kPa.

Rates with strain dependenceMetabolite binding method		(A) P_i_: M1 ATP: M1	(B) P_i_: M2 ATP: M1	(C) P_i_: M1 ATP: M2	(D) P_i_: M2 ATP: M2
(1) None		3.49 %	3.48 %	2.67 %	2.67 %
(2)*k*_1_		3.47 %	3.24 %	2.67 %	2.67 %
(3)*k*_−1_		3.49 %	3.34 %	2.67 %	2.67 %
(4)*k*_2_		2.99 %	2.94 %	2.51 %	2.50 %
(5)*k*_−2_		2.68 %	2.64 %	1.92 %	1.82 %
(6)*k*_3_		3.23 %	3.15 %	2.59 %	2.55 %
(7)*k*_1_ and *k*_−1_		3.49 %	3.24 %	2.67 %	2.67 %
(8)*k*_1_ and *k*_2_		2.99 %	2.94 %	2.51 %	2.50 %
(9)*k*_1_ and *k*_−2_		2.56 %	2.40 %	1.92 %	1.82 %
(10)*k*_1_ and *k*_3_		3.23 %	3.15 %	2.89 %	2.55 %
(11)*k*_−1_ and *k*_2_		2.99 %	2.94 %	2.51 %	2.50 %
(12)*k*_−1_ and *k*_−2_		2.68 %	2.64 %	1.92 %	1.82 %
(13)*k*_−1_ and *k*_3_		3.23 %	3.15 %	2.59 %	2.55 %
(14)*k*_2_ and *k*_−2_		2.42 %	2.41 %	1.75 %	1.72 %
(15)*k*_2_ and *k*_3_		2.99 %	2.94 %	2.51 %	2.50 %
(16)*k*_−2_ and *k*_3_		2.62 %	2.57 %	1.87 %	1.78 %

**FIGURE 6:**
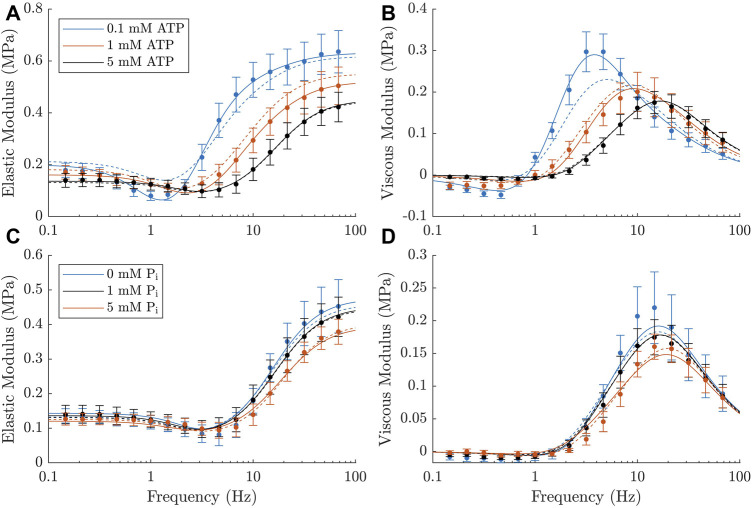
Comparison of the simplest version of the model (1A, dashed lines) and the best fitting model from Table 3 (14D, solid lines). **(A, B)**: Elastic and viscous moduli data at different ATP concentrations compared to the corresponding output of each of the models. **(C, D)**: Elastic and viscous moduli data at different P_i_ concentrations compared to the corresponding output of each of the models. The best-fitting model matches the data better than the simplest version, particularly across the lower ATP concentrations.

Supporting these data, Figure 6 compares the fit obtained using the simplest model with the best fit overall and reveals the key features of the complex modulus that are improved with the more complex model. The most prominent of these areas are the increase in the ‘dip’ magnitude with lower ATP concentrations and the larger effect of decreasing ATP concentration from 1 mM to 0.1 mM. There is also an improvement in the magnitude of the effect of decreasing P_i_ concentration from 1 mM to 0 mM. This best fitting model (14D) has the maximum number of variable parameters, 14. It uses Method 2 to incorporate both ATP and P_i_ and has strain dependence on both *k*_2_ and *k*_−2_

#### 3.3.1 Effect of metabolite binding complexity

The most evident trend relating to the metabolite dependence is that having ATP release in rapid equilibrium (Method 2) offers a much greater improvement in the RMSE than the direct integration to an existing step (Method 1). Under all the types of strain dependence considered in Figure 5, adding *k*_dATP_ to the model (going from blue to green in Figure 5) improves the RMSE by at least 10 % and, in most cases, by over 20 %.

As ATP influences both *k*_−2_ and *k*_3_ under rapid equilibrium, there is a slightly different formulation for $\partial_{x_{C}}^{x_{C}}$ , or the cutoff frequency for $H_{x_{C}}$ , compared to the direct dependence shown in Eq. 32.

$$\partial_{x_{C}}^{x_{C}}=-\frac{k_{-2}^{\prime }k_{dATP}+k_{3}^{\prime }\left[ATP\right]}{\left[ATP\right]+k_{dATP}}$$ (33)

When the model has direct dependence on ATP concentration, $\partial_{x_{C}}^{x_{C}}$ increases linearly with ATP concentration. On a logarithmic scale, this becomes an exponential relationship, which does not support a larger frequency shift between 0.1 mM and 1 mM than between 1 mM and 5 mM (Figure 3). On the other hand, Eq. 33 produces a sigmoidal relationship with ATP on the logarithmic scale which is shifted by *k*_dATP_ and has its steepness determined by the ratio of $k_{3}^{\prime }$ to $k_{-2}^{\prime }$ . These factors allow for a much better fit to our ATP data. Note that $k_{3}^{\prime }$ must be faster than $k_{-2}^{\prime }$ , in order for $\partial_{x_{C}}^{x_{C}}$ to increase with ATP concentration. Under all of the strain dependence locations, the fit is also improved by having the P_i_ release in rapid equilibrium. This suggests that the cutoff frequency shift is also non-linear for this metabolite. The reduced effect in comparison to ATP is to be expected, considering the substantially lower sensitivity of P_i_ across the concentration range we have measured.

#### 3.3.2 Effect of strain dependence location

Our previous work showed that strain dependence in a cross-bridge model augments the minima in the elastic and viscous moduli via *H*_CS_, the strain dependent component of *H*_C_, which took the form of a negative band-pass filter (Musgrave et al., 2022). We posited that this response could be achieved with any strain dependence that reduces the value of *C* with cross-bridge strain and therefore tested all of these options (Table 2). However, the interaction between the rates involved with strain dependence and metabolite concentration resulted in complex effects when trying to fit the model to the magnitude of the ‘dip’, which was not captured by the simplest model. The magnitude of *H*_CS_ is influenced by the partial derivatives relating cross-bridge strain to the proportion of cross-bridges in state **C**. These key values are $\partial_{C}^{x_{B}}$ or $\partial_{C}^{x_{C}}$ , depending on which state’s strain the rate is influenced by (see Table 2).

Strain dependence on *k*_−2_ provided the clearest improvement in the model RMSE (Figure 5). As can be seen in Figure 6, this improvement is driven by the excellent match to the elastic modulus minima across the three ATP concentrations. Strain dependence on *k*_−2_ is able to achieve this because $\partial_{C}^{x_{C}}$ , which is the magnitude of the band-pass transfer function, will always increase with decreasing concentration of ATP:

$$\partial_{C}^{x_{C}}=-C_{0}k_{-2}\phi_{s-2}.$$ (34)

As in Section 3.2, this is driven by the steady-state proportion of cross-bridges in state **C**. When combined with indirect ATP dependence in the model, this provides an even stronger effect as both *C*_0_ and *k*_−2_ increase with lower ATP concentrations.

Strain dependence on *k*_3_ offered only a small improvement in the RMSE compared to no strain dependence at all (Figure 5). The magnitude of *H*_CS_ under this strain dependence takes a very similar form to the *k*_−2_ case $\left(\partial_{C}^{x_{C}}=-C_{0}k_{3}\phi_{s3}\right)$ . However, unlike in Eq. 34, strain dependence on *k*_3_ does not strictly cause an increase in the magnitude of *H*_CS_. This is because, while *C*_0_ increases, *k*_3_ also decreases with lower ATP concentrations. In the best model fits found with this strain dependence, there was a biphasic response where the magnitude of *H*_CS_ was highest at 1 mM of ATP and lower at both 0.1 mM and 5 mM.

Strain dependence on *k*_2_ also improved the RMSE compared to no strain dependence, but only due to some slight improvements in the effect of P_i_. Under this strain dependence the magnitude of *H*_CS_ is proportional to *B*_0_ as $\partial_{C}^{x_{B}}=B_{0}k_{2}\phi_{s2}$ . This means that it will increase with P_i_ and decrease with ATP. The RMSE improvement gained from this strain dependence is mostly due to the slight increase in viscous modulus given by this transfer function, particularly with lower P_i_ concentration.

As for the other transition rates, Figure 5 shows that strain dependencies involving just *k*_1_ or *k*_−1_ do not offer any improvement in the RMSE over the version of the model with no strain dependence. The optimiser attempts to minimise *ϕ*_s_ for these rates because the response of their respective *H*_CS_ functions to metabolite changes opposes the effects needed to improve the fit (Figure 6). Specifically, strain dependence on *k*_1_ causes a similar effect to *k*_2_, but with a much more profound decrease in amplitude with lowered ATP, while strain dependence on *k*_−2_ causes an increase in the amplitude with P_i_ concentration.

### 3.4 Final model

**FIGURE 7:**
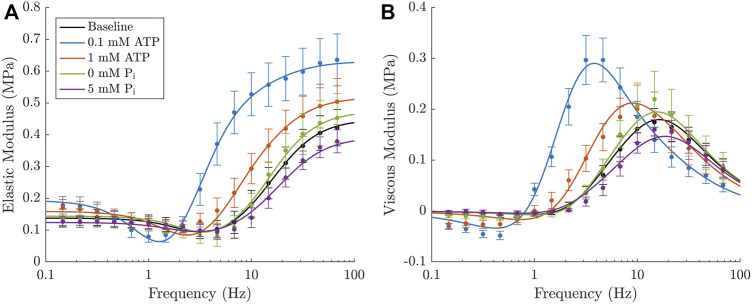
Final iteration of the cross-bridge model which provides the best fit to the experimental elastic **(A)** and viscous **(B)** moduli. The model incorporates both ATP and P_i_ in rapid equilibrium with strain dependence assigned to both *k*_−2_ and *k*_3_ transition rates. A cross-bridge insensitive stiffness was required to fit each of the different metabolite concentrations. The filled circles and error bars represent the mean value ± SEM for each of the five experimental conditions and the respective lines in each colour represent the model prediction under each of these metabolite concentrations.

Our final model (16D) provides an excellent fit to our complex modulus data (Figure 7). The model comprises the baseline equations described in Section 2.2.1 (Eqs. 1–7), a cross-bridge insensitive stiffness (Eq. 22), indirect dependence on ATP and P_i_ as described by Eqs. 16–19 and strain dependence on both *k*_−2_ and *k*_3_ as described in Table 2.

The model has a total of 14 parameters whose values are described in Table 4. Section 3.3 revealed that strain dependence on *k*_−2_ and indirect ATP dependence offered the most significant improvements to the RMSE, so these were the most important elements to include in the final model. We also included the indirect dependence on P_i_ to provide a more accurate description of the non-linear relationship. Strain dependence on *k*_3_ was included, despite only offering a small further improvement in the RMSE, due to the physiological importance of having strain dependence on cross-bridge detachment (see Discussion).

**TABLE 4:** Optimal parameter values of the final model (Model 16D from Table 3).

Parameter	Value	Units
*k*_1_	9.65	s^−1^
$k_{-1}^{\prime }$	19.6	s^−1^
$k_{2}^{\prime }$	13.5	s^−1^
$k_{-2}^{\prime }$	1.11	s^−1^
$k_{3}^{\prime }$	13.0	s^−1^
*ϕ*_x_	9.00	unitless
*ϕ*_v_	0.101	unitless
*ϕ*_l_	3.89	unitless
*K*	3150	GPa⋅m^−1^
*K*_s_	45.0	GPa⋅m^−1^
*ϕ*_s-2_	995	*μ*m^−1^
*ϕ*_s3_	71.1	*μ*m^−1^
*k*_dATP_	2.36	mM
*k*_dPi_	5.00	mM

**FIGURE 8:**
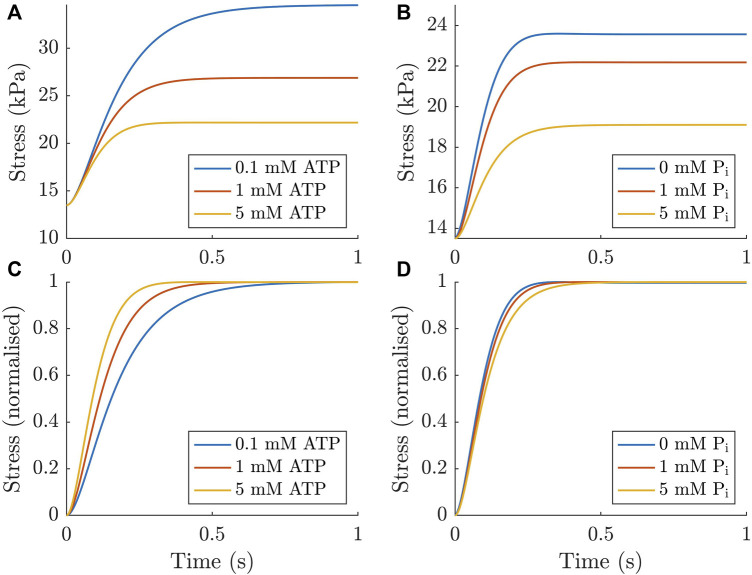
Force redevelopment simulated numerically in the ODE model under different concentrations of ATP and P_i_. These simulations were performed by enforcing a short period of detachment by altering the cycling rates to encourage detachment for 10 ms (similar to Rice et al. (2008)). As expected, increasing the concentration of both metabolites reduces the stress generated **(A, B)**. When normalised to the force developed, increasing ATP concentration increases the rate of force redevelopment **(C)**, while increasing P_i_ concentration has the opposite effect **(D)**.

The force redevelopment simulations from the final model show that the model produced steady-state stresses that fell within the standard error of the means presented in Section 3.1 and exhibits typical behaviour in the time domain (Figure 8A, B). The lower panels (Figure 8C, D) show the more commonly reported normalised force redevelopment as a function of either ATP or P_i_. These plots indicate that there was an increased rate of force redevelopment with increasing ATP concentration and a decreased rate of force redevelopment with increasing P_i_ concentration. Both of these trends agreed with those found in the Tewari et al. (2016) study and the simulated effect of ATP was also consistent with experimental data from Ebus et al. (2001).

## 4 Discussion

In this study, we explored the effects of metabolite binding and their interaction with strain dependence on the frequency response of cardiac muscle. We showed that the experimentally observed effects of ATP and P_i_ concentration on the cardiac complex modulus can be attributed to their regulation of cross-bridge detachment rates. The results from our linear analysis of the cross-bridge model reconcile, and provide a mechanistic basis for, the biochemical schemes which place P_i_ release following cross-bridge formation and ATP binding prior to cross-bridge detachment. When the complexity of metabolite binding and its interaction with strain dependence was explored in a systematic manner, assessing 64 model permutations, we identified the key mechanisms which produced the best model fit to our experimental data. Analysis of these results led to the identification of a final model that produced an excellent fit to the data, and incorporates well-justified mechanisms for metabolite and strain dependencies.

### 4.1 Selection of the final model

Following the systematic model fitting of different permutations of the cross-bridge model to our data, we have selected one of the most complex versions of the model, with 14 variable parameters, as our final model. Given our objective of finding the most parsimonious model which captures cross-bridge level kinetics, the reader may be wondering why a simpler model was not chosen. If the sole purpose of the model was to recapitulate the experimental data we had measured, Model 5C - a 12-parameter model which only used Method 2 for ATP and only had strain dependence on *k*_−2_ - may have been the most appropriate. However, we are also interested in applications of this model outside of the specific measurements it was calibrated under. It was for this reason that we chose to include the *k*_d_ parameter for P_i_ and use Method 2 for both metabolites. Because our data showed relatively low sensitivity to P_i_ concentration, this offered a much smaller improvement in the RMSE, but Method 2 still captures the non-linear frequency behaviour better than the alternative. The ability to provide a more accurate representation of the P_i_ dependence will be important when considering altered metabolic states of heart tissue under disease conditions. For example, this will be beneficial for understanding implications of diabetes or myocardial ischemia on the heart, which are both associated with elevated P_i_ concentrations (Terkildsen et al., 2007; Valkovič et al., 2022).

The parameter which offered the smallest improvement was *ϕ*_s3_, accounting for strain dependence on *k*_3_. While it is evident that this strain dependence does not make a large contribution to recapitulation of the complex modulus, we have chosen to keep this as it is considered an essential element of cross-bridge theory. This component reflects the concept of strain causing the detachment of cross-bridges, which dates back as far as Huxley’s seminal model (Huxley, 1957).

### 4.2 Mechanisms underlying metabolite dependence in the model

The linearisation analysis we have completed offers deeper insights into the mechanisms whereby metabolite concentrations affect the mechanical response of cardiac muscle, as well as more general insights into cross-bridge physiology which support conclusions from our previous work (Musgrave et al., 2022). A visual summary of each of the transfer functions which make up the full complex modulus response and their response to metabolite changes is presented in Appendix B.

The key effects of decreases in P_i_ and ATP concentrations on the complex modulus, namely, the increase in stiffness at both low and high frequencies and the leftward shift in frequency, arise directly from slower detachment rates in the cross-bridge cycle. As presented in Section 3.2, the slow detachment via either *k*_−1_ or *k*_3_ results in more cross-bridges attached in the steady state of the post-power stroke state, *C*_0_. This mechanism is responsible for the increased stiffness and higher steady-state force. Slower detachment rates also affect the cutoff frequencies of the transfer functions representing stiffness coming from the strain of attached cross-bridges, $H_{x_{B}}$ and $H_{x_{C}}$ , which are responsible for the frequency shift seen in the data. Reduced detachment rates correspond to cross-bridges staying attached for longer, allowing time for strain development and increased stiffness at lower frequencies.

The differences between the two methods of implementing metabolite dependence are subtle enough to go unnoticed without closer inspection. The direct dependence (Method 1) of either ATP on *k*_3_ or P_i_ on *k*_−1_ produces a linear relationship between the metabolite and the cutoff frequency for $H_{x_{B}}$ or $H_{x_{C}}$ , which does not reflect what is observed in the data. The relationship is better captured using rapid equilibrium (Method 2) which requires an additional parameter, *k*_d_. This implementation does, however, introduce limits on the values of *k*_2_ and *k*_−2_ (for P_i_ and ATP, respectively) because of their close association with the two detachment rates. A positive relationship between metabolite concentration and frequency, as observed experimentally, will occur only when $k_{-1}^{\prime }$ is larger than $k_{2}^{\prime }$ and $k_{3}^{\prime }$ is larger than $k_{-2}^{\prime }$ . This is not always the case in parameterisations of similar published models (Rice et al., 2008), but has not been a limitation in fitting to the data presented here.

In terms of the mechanisms underlying these two different methods, the direct dependence (Method 1) of a metabolite assumes that the binding or release of ATP or P_i_ occurs simultaneously with a mechanical state change in the acto-myosin filaments. On the other hand, the indirect dependence (Method 2) assumes that the binding or release occurs in a separate, but more rapid step, which occurs between the major mechanical transitions. More complex myosin kinetic schemes do not always separate ATP binding and cross-bridge detachment, instead combining them into a single step (Houdusse and Sweeney, 2016; Offer and Ranatunga, 2020; Moretto et al., 2022). Fitting of our results provides strong evidence that these steps are, and should be treated as, separate. Further evidence for our decision to use Method 2 for P_i_ comes from a much stronger consensus that P_i_ release occurs following the initial cross-bridge formation, rather than concurrently (Houdusse and Sweeney, 2016; Walklate et al., 2016; Woody et al., 2019; Offer and Ranatunga, 2020; Moretto et al., 2022). There is, however, still debate around whether P_i_ release occurs before or after the power stroke. From single molecule measurements Woody et al. (2019) recently showed that P_i_ release occurs after the cross-bridge power stroke. However, simulations of this configuration produce velocity-P_i_ and force-P_i_ relations that are inconsistent with experimental observations (Offer and Ranatunga, 2020). In our simplified model, placing the P_i_ release in this position causes complete inversion of the force-P_i_ relationship and so our final placement is the only feasible option for the three-state model. Recently, a study from (Moretto et al., 2022) using single molecule fluorescence, high-speed atomic force microscopy and molecular modelling proposed a complex model which incorporates a multistep P_i_ release mechanism, to reconcile these contradictions.

### 4.3 The effect of strain dependence in the model

While previous work found that each of the transition rates can contribute to the strain-dependent response required for the cardiac complex modulus (Musgrave et al., 2022), in this study we have found that this behaviour was dependent on metabolite concentrations. In order to fit the cross-bridge models to our data we had to find a strain dependence placement which increased the magnitude of *H*_CS_ with ATP. This was found to only be possible with strain dependence on *k*_−2_. In our systematic analysis, strain dependence at *k*_−2_ was a key mechanism required for producing good model fits to the data. Through this mechanism, strain in state **C** decreases the number of cross-bridges in the post-power stroke state by increasing the reverse power stroke transition rate, to a greater extent under low ATP conditions. A similar strain dependence on *k*_−2_ was suggested by Rice et al. (2008) as an alternative way to simulate the increase of isomerisation rates under muscle shortening and *vice versa* in order to produce sufficient shortening velocities. Tewari et al. (2016) also had asymmetrical strain dependence with a similar effect, albeit on a four state model.

While it did not induce as large an improvement on the RMSE as *k*_−2_, we also included strain dependence on *k*_3_, the forward detachment rate. As described in Section 4.1, this is the fundamental strain-dependent rate from the Huxley (1957) model and thus strain dependence on this rate is ubiquitous among existing models (Razumova et al., 1999; Rice et al., 2008; Tewari et al., 2016; Land et al., 2017; Musgrave et al., 2022). This strain dependence increases cross-bridge detachment with increasing strain, meaning that there is a reduced number of cross-bridges in state **C** under higher strains. Like the strain dependence on *k*_−2_, this contributes to the characteristic dip in the elastic modulus. However, the effect of ATP concentration on this behaviour is not consistent. We found the response of *H*_CS_ to be biphasic in response to ATP concentration when strain dependence was placed on *k*_3_. This was due to opposing effects on the key partial derivative for this transfer function, originating from the ATP dependence on *k*_3_ itself and on *C*_0_.

Our previous work touched on the relevance of the specific form of the strain-dependent relationship when reproducing complex moduli (Musgrave et al., 2022). A limitation of the linearisation method is that any strain-dependent rate function with a derivative that is undefined or has a value of zero at its equilibrium can not be used in the Taylor series expansion and thus will not contribute to *H*_CS_, the strain-dependent transfer function. More generally, however, a purely symmetrical rate function will also not produce any strain-dependent effect on the complex modulus. We have therefore used exponential functions to satisfy both of these requirements, which has been used previously in a model fitted to complex moduli, (Tewari et al., 2016). However, there may be an additional symmetrical component to the cross-bridge strain-dependence which induces detachment following both lengthening and shortening of the muscle, which can not be captured from the complex modulus measurement. This could explain why strain dependence on *k*_3_, which is otherwise assumed to be crucial, had such a small effect on our model fitting.

### 4.4 Cross-bridge insensitive stiffness in activated muscle

An addition to our model that differs from previous work was the cross-bridge insensitive stiffness (Eq. 22). This component proved to be essential for fitting the metabolite-sensitive active complex modulus data. In the absence of this component, the goodness of fit achieved by the best permutation of the model was a normalised RMSE of 3.54 % (worse than the simplest model in Table 3). This is because the changes in *C*_0_ required to reproduce the measured changes maximum elastic modulus or the change in steady-state stress with metabolites resulted in much larger changes in the static modulus than what was measured. Since all of these components reflect the number of cross-bridges bound in the post-power stroke state, this implies that there was a component to the static modulus of the muscle which did not relate to the proportion of attached cross-bridges. Importantly, this is distinct from what are traditionally considered to be ‘passive’ properties of the muscle (Maughan et al., 1998) because the active complex modulus was found by subtracting the complex modulus measured under relaxed (pCa 9.0) conditions. Therefore, this stiffness is only present under high Ca^2+^ and can be elucidated only by performing an intervention that affects the number of cross-bridges attached while the muscle is fully activated. While we have implemented this as a phenomenological element in the model, a proposed mechanism underlying this effect may be the influence of Ca^2+^ on titin stiffness, as has been clearly shown in skeletal muscle (Nishikawa et al., 2020), and to a more limited extent in cardiac muscle (Fujita et al., 2004). Further investigation may be required to determine if this phenomenon is unique to permeabilised muscle and how it may influence the behaviour of intact muscle if it is not.

### 4.5 Is cross-bridge cycling faster with higher concentrations of ATP or P_i_?

The analysis we have performed in this study has provided some clarity around the above question. As discussed in Section 4.2, the shift toward higher frequencies in the complex modulus is driven by increased detachment rates under higher metabolite concentrations. Upon making some of the earliest measurements which observed this phenomenon, Kawai et al. (1993) suggested that this frequency shift was related to faster cross-bridge kinetics at higher ATP concentrations. While, in the case of increased ATP and P_i_, there are faster rates associated with the higher metabolite concentration, we question if this is the most intuitive understanding of the underlying processes.

Another common way to assess the rates of cross-bridge cycling is by measuring *k*_tr_, the rate of force redevelopment following a rapid length change (Brenner and Eisenberg, 1986). Experimental measurements have shown that cardiac force redevelopment rates are faster under elevated ATP (Ebus et al., 2001) and P_i_ concentrations (Hinken and McDonald, 2004). However, an important caveat in these results is that the force was normalised, as in Figure 8C, D. Based on the mechanisms in the model, and the fundamental principles of biochemistry, both metabolites should in fact reduce the true rate at which force is developed (they both slow down the rate at which cross-bridges accumulate in state **C**). This result is evident from our non-normalised plots (Figure 8A, B). We are not suggesting that there is no value in normalising the force redevelopment; our normalised results clearly show that, while force redevelopment is slower at high ATP concentrations, the muscle does reach its steady-state force more quickly with higher ATP concentration (Figure 8C). However, these seemingly contradictory results serve as a reminder to be cognisant of the underlying mechanisms when interpreting normalised data.

### 4.6 ADP

Due to a lack of data describing the effect of ADP, we have not included this metabolite in the model analysed for this study. However, due to the known effect of force increase with ADP concentration (Godt and Nosek, 1989), there is only one plausible way to incorporate ADP dependence. As described by Tran et al. (2010), this metabolite must be placed in a rapid equilibrium step state **C** to reproduce this effect in a three-state model. This would result in effects that are generally in opposition to the ATP effects explored in previous sections.

### 4.7 Closing remarks

Our findings from linearising a metabolite-sensitive ODE-based cross-bridge model will help to inform considered future development of models of this type. While the model we have presented has been parameterised for rat cardiac trabeculae, the linearisation approach facilitates further parameterisation with other data sets. Given that some of the model choices we made could have been to aid in fitting our particular data set, it is plausible that future modellers may be interested in considering some of the permutations of the model which we explored as part of their parameterisation process. Code for fitting the model, along with a flexible version of the model which allows these different options to be considered is available at https://github.com/JuliaMusgrave/XBModel_2024_Rat.

## Figures and Tables

**FIGURE B1:**
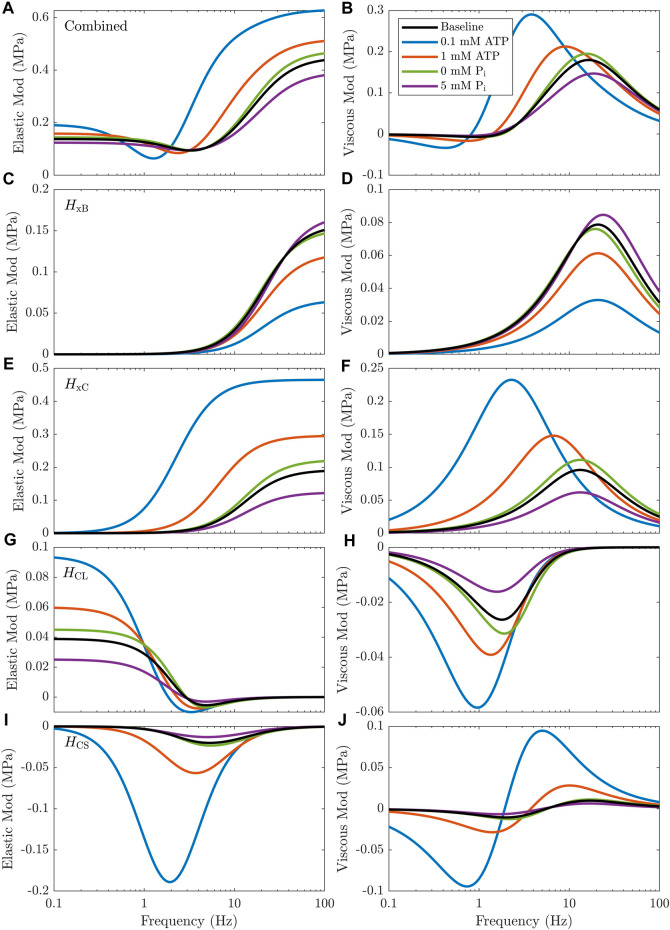
Effect of changing metabolite concentrations on individual transfer functions in the final model.

## Data Availability

The raw data supporting the conclusion of this article will be made available by the authors, without undue reservation.

## Appendix A: Full model equations

Active muscle force:

$$F=K\left(Bx_{B}+Cx_{C}\right)+F_{s}$$

Cross-bridge insensitive component of force:

$$F_{s}=\left\{\begin{array}{cc} K_{s}\left(L-1.9\right)\; & L>1.9,\\ 0\; & L\leq 1.9, \end{array}\right.$$

Proportion of cross-bridges in each state:

$$\frac{dB}{dt}=k_{1}A-\left(k_{-1}+k_{2}\right)B+k_{-2}C$$

$$\frac{dC}{dt}=k_{2}B-\left(k_{-2}+k_{3}\right)C+k_{-3}A$$

$$A=Z\left(L\right)-B-C$$

Dependence of total available cross-bridges on sarcomere length:

$$Z\left(L\right)=\left\{\begin{array}{cc} 1+\phi_{l}\left(\frac{L}{L_{max}}-1\right)\; & L<L_{max},\\ 1\; & L_{max}\leq L\leq 2.4, \end{array}\right.$$

Cross-bridge strain/distortion in each attached state:

$$\frac{dx_{B}}{dt}=-\frac{\phi_{x}}{B}\left(k_{1}A+k_{-2}C\right)x_{B}+\phi_{v}\frac{dL}{dt}$$

$$\frac{dx_{C}}{dt}=-\frac{\phi_{x}}{C}\left(k_{2}B+k_{-3}A\right)\left(x_{C}-x_{C0}\right)+\phi_{v}\frac{dL}{dt}$$

Metabolite-dependent transition rates:

$$k_{-1}=k_{-1}^{\prime }\frac{\left[P_{i}\right]}{k_{dPi}+\left[P_{i}\right]}$$

$$k_{2}=k_{2}^{\prime }\frac{k_{dPi}}{k_{dPi}+\left[P_{i}\right]}$$

Metabolite-dependent and strain-dependent transition rates:

$$k_{-2}=k_{-2}^{\prime }\frac{k_{dATP}}{k_{dATP}+\left[ATP\right]}e^{\phi_{s-2}\left(x_{C}-x_{C0}\right)}$$

$$k_{3}=k_{3}^{\prime }\frac{\left[ATP\right]}{k_{dATP}+\left[ATP\right]}e^{\phi_{s3}\left(x_{C}-x_{C0}\right)}$$
